# Cognitive impairment in essential tremor assessed by the cerebellar cognitive affective syndrome scale

**DOI:** 10.3389/fneur.2023.1224478

**Published:** 2023-08-18

**Authors:** Virginie Destrebecq, Gilles Naeije

**Affiliations:** ^1^Clinique Universitaire de Bruxelles (CUB) Hôpital Erasme, Department of Neurology, Université Libre de Bruxelles, Brussels, Belgium; ^2^Laboratoire de Neuroanatomie et Neuroimagerie translationnelles, UNI-ULB Neuroscience Institute, Université Libre de Bruxelles, Brussels, Belgium

**Keywords:** cerebellar cognitive affective syndrome, essential tremor, CCAS-Scale, Purkinje cell, cognitive disorder

## Abstract

**Background:**

Essential tremor (ET) is a movement disorder characterized by cerebellar neurodegenerative changes. ET is also associated with non-motor symptoms including cognitive impairment. The neuropsychologic profile of a patient with ET could relate to cerebellar cognitive affective syndrome (CCAS).

**Objective:**

This study aimed to assess the prevalence of cognitive impairment in patients with ET and identify whether the cognitive impairment in ET corresponds to a CCAS.

**Methods:**

Cognitive functions were evaluated with the CCAS-Scale (CCAS-S) in 20 patients with ET and 20 controls matched for age, sex, and level of education. The results of the CCAS-S were compared between patients and controls. The underlying determinant of CCAS inpatients with ET was identified through the correlation between the results of the CCAS-S and age at onset of symptoms, disease duration, and the Essential Tremor Rating Assessment Scale (TETRAS).

**Results:**

On a group level, ET patients performed significantly worse than matched controls. In total, 13 individuals with ET had a definite CCAS (CCAS-S failed items ≥ 3). ASO and TETRAS scores significantly correlated with CCAS-S performances in ET patients.

**Conclusion:**

CCAS is highly prevalent in patients with ET which supports the cerebellar pathophysiology of associated cognitive impairment and supports a more systematic use of the CCAS-S to cognitively assessed patients with ET.

## 1. Introduction

Essential tremor (ET) is a common neurologic disorder whose prevalence increases from 0.04% in the population under 20 years to 3% above aged 80 years (1). The cardinal symptom of ET is an action tremor that mainly affects the upper limbs but can also involve the head, voice, and other body regions (2, 3). Pathological studies, disease course, and age-related prevalence provide evidence that ET is a cerebellar neurodegenerative disease associated with morphologic changes centered around the Purkinje cells (PC) of the cerebellar cortex with heterotopic PC, PC loss, PC axonal swelling, and redistribution of climbing fiber synapses to the outer PC dendritic arbor (4). Conventional structural imaging does not disclose a specific pattern of atrophy neither in the cerebellum nor in the hemispheric cortices, but diffusion tensor imaging (DTI) consistently shows across studies of microstructural alterations of the cerebellar peduncles and the dentate nuclei that suggests an impairment of the cerebellar afferent and efferent tracts involved in the cerebello-cortical loops (5). These microstructural alterations have functional repercussions in functional magnetic resonance imaging (fMRI), altered cerebello-thalamo-cortical resting-state functional connectivity, as well as functional connectivity alterations within the sensorimotor and fronto-parietal resting-state networks (RSN) and the default mode network (DMN) (6–8). Historically considered as benign monosymptomatic affection, the understanding of ET impact has considerably evolved along with amounting pathological and functional imaging evidence. Indeed, the cortico-cerebellar loops affected in ET, in addition to motor control, are involved in many perceptual and cognitive processes (9, 10) which may explain why non-motor symptoms are increasingly identified in ET including mood disorders and cognitive impairment (11, 12). In view of these pathological (4) and neuroimaging (5, 7–9) abnormalities in ET patients involving the cortico-cerebellar loops, the cognitive disorders observed in these patients could be related to a disruption of this cortico-cerebellar tract. However, individuals with ET, compared to healthy individuals, perform within the normal ranges on the screening tools commonly used to detect cognitive abnormalities, such as the Mini-Mental State Evaluation (MMSE) and the Montreal Cognitive Assessment (MOCA) with similar results for the MOCA and 1.16 point less on the MMSE (13). However, when more comprehensive neuropsychological test batteries are used, individuals with ET have lower performances in attention, executive functions, verbal memory, verbal fluency, category-switching fluency, and mental set-shifting than matched controls (14–16). This combination of relatively mild but global higher neocortical dysfunction is characteristic of the cerebellar cognitive and affective syndrome (CCAS). The CCAS is the cognitive counterpart of movement dysmetria and lack of accuracy in cerebellar diseases and typically combines language, emotion regulation, memory, attention, visuospatial, and executive functions (17, 18). A CCAS screening and follow-up scale (CCAS-S) was designed in 2018 based on the neuropsychological tests that could most efficiently single out individuals with cerebellar pathology from healthy individuals and includes 10 items with a raw and passing score: a semantic fluency task, a phonemic fluency task, a verbal category switching task, a forward and backward digit span, a cube drawing task, a verbal registration task, a verbal similarities task, a Go No-Go task, and affect evaluation (17). The CCAS-S showed a high yield to detect CCAS in patients with both acquired and genetic cerebellar disorders (17). Strikingly, in the cohorts that validated the CCAS-S, the patient presented the same combination of normal MMSE and MOCA and failed specific neuropsychological tests than individuals with ET (17). Conversely, to full neuropsychological test batteries that take hours to fulfill, the CCAS-S has the major advantage to be a paper and pencil test that can be performed at the bedside in ~10 min. Since its validation in 2018, the CCAS-S has been used to screen for CCAS in several kinds of cerebellar diseases such as spino-cerebellar ataxias (SCA) 2, 3, and 6, cerebellar strokes, multiple system atrophy type C (MSA-C), and Friedreich ataxia (FA). In those studies, the CCAS-S consistently described poorer performances in patients with cerebellar diseases (19–23).

In this study, we postulate that cognitive impairment in ET may correspond to a CCAS that can be identified by the CCAS-S. To test that hypothesis, we evaluated 20 individuals with ET with the CCAS-S and sought for correlation between age at symptom onset (ASO), disease duration, ET clinical characteristics, and the CCAS-S performance that would support a link between ET severity and cognitive impairment magnitude.

## 2. Materials and methods

### 2.1. Patients

This is a monocentric observational study that included 20 (nine female and 11 male) patients with ET followed at the CUB-Hôpital Erasme, Brussels (Belgium), and 20 controls matched for age, gender, and level of education. All participants contributed to the study after written informed consent and prior approval of the study by the CUB Hôpital Erasme Ethics Committee (CCB: B406201941751).

The diagnosis of the essential tremor was made by two neurologists experienced in movement disorders. Patients fulfilled the criteria for essential tremor defined by the Tremor Task Force of the International Parkinson and Movement Disorder Society (24). The exclusion criteria for participation were chronic diseases affecting the nervous system, psychiatric disorders, chronic alcohol abuse, and the use of drugs and psychotropic molecules including antiepileptic treatments.

### 2.2. Clinical evaluation

The clinical cognitive assessment was performed with the version 1A of the CCAS-S: a mean raw score was calculated for each of the 10 items which composed the scale (semantic fluency task, phonemic fluency task, verbal category switching task, forward and backward digit span, cube drawing task, verbal registration task, verbal similarities task, Go No-Go task, and affect evaluation) (12). A raw score is obtained for each task, with a minimum passing score. The number of failed tests determines the likelihood that the subject has CCAS: Three or more failed tasks make a definite CCAS.

The severity of the tremor and its impact on daily lives have been assessed through the Essential Tremor Rating Assessment Scale (TETRAS) developed in 2003 by the Tremor Research Group (TRG) which has been recognized as a reliable tool for the clinical assessment of essential tremor (25, 26). This scale is composed of a performance section composed of nine items (head tremor, face tremor, voice tremor, upper limb tremor, lower limb tremor, spiral drawing, handwriting, standing, and a DOT approximation task) for a maximum total score of 64 and activities of daily live section (ADL) composed of 12 items supposed to cover daily live activities and social impact for a maximum total score of 48 [For details see (25, 27)]. A high total reflects a severe tremor that adversely affects ADL.

### 2.3. Statistical analysis

Differences between groups were assessed using Student's *t*-tests (two-tailed) for the CCAS-S raw score and the number of failed tests.

In ET patients, Spearman's rank correlation tests were used to assess possible relations between the CCAS-S raw score and clinical features of the patients, including age, ASO, disease duration, the total performance score, and the total ADL score of the TETRAS. Bonferroni correction for multiple comparisons (*n* = 3) was used and led to significance at a *p*-value of < 0.017. All analyses were performed using JASP.

## 3. Results

The demographic characteristics of patients and healthy individuals were comparable and are summarized in Table 1.

**Table 1:** Demographic and clinical data.

	**Patients with ET (20)**	**Healthy controls (20)**
Mean age (±SD), years	65 ± 13	62 ±1 3
Male	11 (55%)	11 (55%)
Female	9 (45%)	9 (45%)
Educational years	14.28 ± 2.04	14.30 ± 2.20
Mean age at onset (±SD), years	50 ± 18	NA
Mean disease duration (±SD), years	14 ± 10	NA
Mean total performance score TETRAS (±SD)	15.26 ± 4.81	NA
Mean total ADL score TETRAS (±SD)	14.84 ± 4.67	NA

SD, Standard deviation; NA, not applicable; TETRAS, The Essential Tremor Rating Assessment Scale; total performance score of 64 and ADL score of 48. A higher score corresponds to a more severe clinical impact.

The mean CCAS-S raw score was significantly lower in patients with ET compared to the healthy individuals (85 ± 15 in individuals with ET vs. 115 ± 10 in controls, *p* < 0.001) and patients with ET failed on average more test items (3.36 ± 2.45) compared to matched controls (0.28 ± 0.41), *p* = 0.01. In total, 13 individuals with ET displayed a definite CCAS (≥3 failed items), and 15 patients failed at least one item while only five controls had a possible CCAS by failing one item out of 10.

The results of the CCAS-S evaluation are detailed in Table 2. In patients with ET, the most often failed items were, by order of frequency, as follows: fluency tasks (phonemic and category switching tasks were more affected than semantic fluency), digit span, and regulation.

**Table 2:** Neuropsychological results.

	**ET patients**		**Healthy controls**		**Passing score/Maximum score**
	**Mean raw score** ±**SD**	**Number of subjects underpassing test (%)**	**Mean raw score** ±**SD**	**Number of subjects underpassing test (%)**	
Semantic fluency^a^	16.9 ± 5.1	7 (35%)	28.9 ± 3.3	0 (0%)	16/26
Phonemic fluency^a^	10.3 ± 4.1	9 (45%)	18.1 ± 5.3	1 (0.05%)	10/19
Category switching^b^	10.7 ± 2.3	9 (45%)	17.6 ± 2.9	0	10/15
Digit span forward^c^	5.6 ± 0.9	9 (45%)	6.7 ± 0.9	1 (0.05%)	6/8
Digit span backward^c^	3.9 ± 0.6	7 (35%)	5 ± 0.28	1 (0.05%)	4/6
Cube drawing/copy	12.5 ± 2.5	5 (25%)	15 ± 0	0 (0%)	12/15
Verbal recall	11.8 ± 2.8	7 (35%)	13.29 ± 1.39	0 (0%)	11/15
Similarities	6.4 ± 1.4	7 (35%)	8 ± 1.1	0 (0%)	7/8
Go No-Go	1.4 ± 0.7	3 (15%)	1.86 ± 0.24	0 (0%)	1/2
Affect^d^	3.2 ± 1.6	10 (50%)	5.57 ± 0.49	1 (0.05%)	5/6
Total	85 ± 15	3.4 ± 2	115 ± 10.29	0.28 ± 0.41	82/120

SD, Standard deviation. ^a^Number of correct words, ^b^number of correct alternations, ^c^correct numbers of a series, and ^d^number of non-affected items.

**Figure 1:**
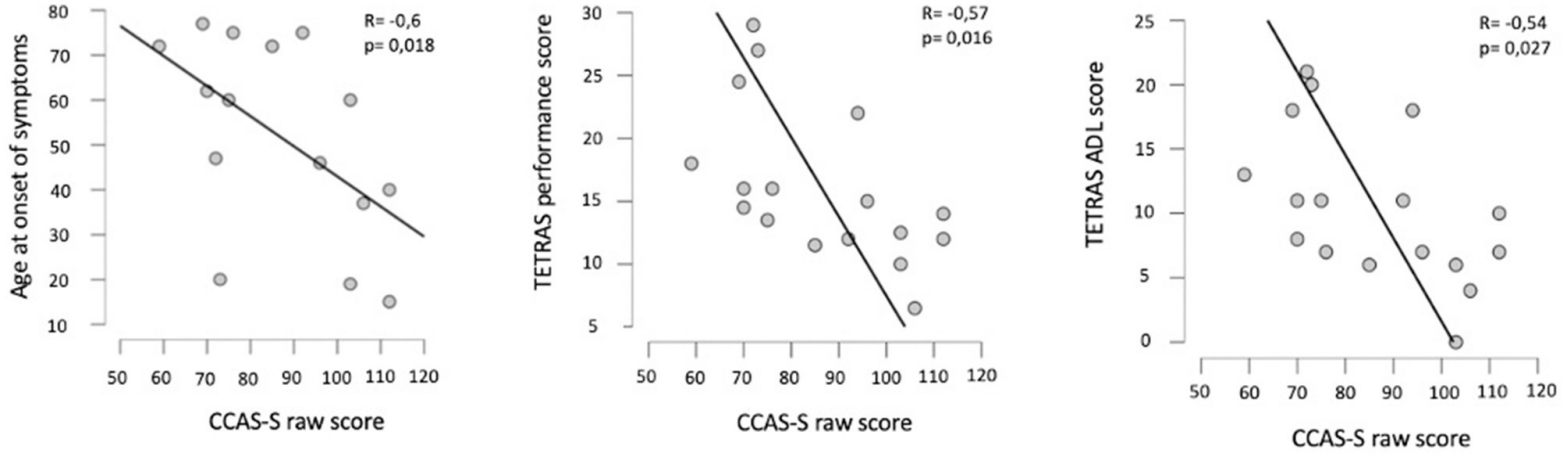
Plots of correlation between the age at onset of symptoms and the CCAS-S raw score **(left)**, between the TETRAS performance score and the CCAS-S raw score **(middle)**, and also between the TETRAS ADL score and the CCAS-S raw score **(right)**. *R*, Spearman's correlation coefficient; p, probability of Spearman's rank correlation test.

We found a statistically significant negative correlation between the CCAS-S raw score and the ASO. The severity of the tremor reflected by the TETRAS was also correlated with the CCAS-S raw score and has a negative impact on the neuropsychological results. These results remained significant after Bonferroni correction for the ET clinical severity and not significant for ASO (*p* = 0.018). Figure 1 illustrates the correlation plots. The results of the Spearman rank correlation tests are summarized in Table 3.

**Table 3:** Correlation with the CCAS-S performances.

	**Correlation with CCAS-S raw score**	
	**Coefficient**	***p***
Age at onset of symptoms	−0.60	0.018
Disease duration	0.046	0.87
TETRAS performance score	−0.573	**0.016**
TETRAS ADL score	−0.535	**0.027**

The bold character indicates the statistically significant *p*-value of Spearman's rank correlation test. Bonferroni corrected *p*-values for multiple comparisons are significant < 0.017.

TETRAS, The Essential Tremor Rating Assessment Scale.

## 4. Discussion

The main findings of this study are that CCAS is highly prevalent in individuals with ET compared to matched healthy individuals and that its severity is correlated with both age of symptoms onset and ET severity.

Despite our relatively small sample of individuals with ET, these results are likely to reflect the situation of individuals with ET in general. Indeed, our cohort shares the same characteristics in terms of age, sex, and disease duration compared to the previous cohorts that evaluated cognition in ET (16, 27, 28).

Patients with ET displayed strikingly poorer performances in the CCAS-S than healthy individuals matched for age, sex, and level of education: two-thirds of ET patients showed a definite CCAS, and three-quarters failed at least one item compared to healthy individuals that failed at most one item and only in five individuals. This study highlights both the high prevalence of cognitive difficulties in ET and the yield of the CCAS-S to detect them as well as to discriminate individuals with ET from healthy controls. The fact that most patients failed one or more CCAS-S items falls in line with a 2022 narrative review in which the 11 studies that were reported used dedicated neuropsychological test batteries to evaluate cognition in ET and found poorer performances in these patients (14). Those facts argue for a more systematic use of the CCAS-S as a screening and evaluation tool for cognitive symptoms in ET. However, compared to the population with other degenerative cerebellar ataxia, individuals with ET seem to display a milder form of CCAS. In studies that assessed CCAS in cerebellar disorders, the lowest pathological CCAS-S raw score was found in patients with MSA-C and SCA2, ~60 (18) and 70 (21), respectively. On the other end of the cognitive impairment range, patients with SCA3 and FA tended to have a more limited impairment close to the values we found in our individuals with ET (25, 29). Interestingly, this spectrum of severity from the milder form of CCAS in ET and SCA3 to more severe forms in SCA2 and MSA-C also corresponds to the severity spectra of the cerebellar cortex degenerative changes, centered around PC cells (PC loss, heterotopic PC, PC dendritic, and axonal and synaptic changes) described on pathology in SCA2, MSA-C, SCA3, FA, and ET (4). Those findings on pathology add to the facts that in MSA-C and SCA2 (30, 31), cerebellar, cerebellar peduncle atrophy (32–34), and microstructural damages are more prominent than in SCA 3 and ET (5). These facts suggest that CCAS severity depends on cerebellar cortical and cerebello-cortical loop integrity. Under the dysmetria of thought framework (35), the CCAS is thought to build up from the disconnection of neocortical areas involved in cognitive processes and the cerebellum. The CCAS, thus, corresponds to a functional cerebello-cortical diaschisis (CCD) (29, 36, 37). Interestingly, this could also explain why the neuropsychological profile of our individuals with ET is largely dominated by an impairment of (phonemic > semantic) verbal fluency, digit span difficulties, and affect dysregulation. Attention, executive functions, and mood rely on frontal lobe function. Therefore, the cognitive abnormalities in patients with ET in the domains that rely on frontal cortex integrity parallel the metabolic brain functional imaging studies on CCD that showed that the frontal cortex was the most metabolically impaired cortical area after a cerebellar lesion (38, 39). In individuals with ET, cortical diaschisis from the cerebello-cortical loop could also explain brain PET-FDG studies that found a cortical glucose hypometabolism occurring mainly in the cortical area densely targeted by cerebellar output tracts (40, 41). Areas that also develop atrophy proportionally to cerebellar stroke volume in stroke contexts (39). In ET, the tremor is thought to relate to a loss of PC inhibition on the dentato-thalamo-cortical tract (DTC) and deep brain stimulation (DBS) that targets the thalami and severe DTC improves tremor severity (42, 43). The CCAS observed in our cohort of individuals with ET is also likely caused by similar excessive activity of the DTC tracts. In future studies, the evaluation of the CCAS-S before and after a thalamic DBS intervention could bring further evidence for the relationship between CCAS and cerebello-cortical loops in individuals with ET if the CCAS-S improves after surgery.

Finally, the inverse relation between poorer performance in the CCAS-S of patients with ET and late ASO is in line with previous studies that associate later onset compared to ET with more severe kinetic tremor of upper limbs (44), the presence of head and voice involvement (45), and cognitive and psychiatric disorder (45). Similarly, the correlation between ET clinical severity assessed by TETRAS and CCAS-S further highlights the link between more severe cerebellar pathology and worse CCAS-S. Indeed, pathological and neuroimaging studies have shown that the extent of cerebellar atrophy (46), the loss of cerebellar gray matter density (47), and pathological changes in the vermis [49] are greater in ET cases with severe and extended tremor including head and voice tremor than with isolated upper limbs tremor.

The main limitation of this study is its small sample size which makes its results preliminary and will have to be confirmed in a larger cohort of patients. The lack of systematic evaluation of depression and other psychiatric symptoms apart from the affected item of the CCAS-S does not exclude an intermingling between mood disorders and cognitive impairments in an individual with ET. This caveat warrants further investigations to weigh the impact of mood disorders associated with chronic pathology from the specific impact of cerebellar pathology in ET cognitive symptoms. However, our results are consistent with the dysmetria of thought framework of CCAS in cerebellar diseases and fit with the functional imaging data obtained in ET as well as with the pathological findings. All these suggest that cognitive impairments are highly prevalent in individuals with ET and find their origin in the cerebellum of individuals with ET. This study also argues for a more systematic use of the CCAS-S for screening and identifying cognitive impairment in individuals with ET. A better understanding of the scope of cerebellar cognitive impairment in individuals with ET could help improve the care of individuals with ET and include tailored cognitive assessment in DBS interventions.

## Data availability statement

The raw data supporting the conclusions of this article will be made available by the authors, without undue reservation.

## Ethics statement

The studies involving human participants were reviewed and approved by CUB Hôpital Erasme Ethics Committee. The patients/participants provided their written informed consent to participate in this study.

## Author contributions

VD and GN: research design, analysis and interpretation of the data, and drafting of the manuscript. VD: acquisition of the data. All authors: revision of the manuscript. All authors have made substantial contributions to the presented study, read, and approved the final submitted manuscript.
